# Professional Autonomy as a Protective Factor Against Burnout: Important but Not Sufficient

**DOI:** 10.7759/cureus.112006

**Published:** 2026-07-03

**Authors:** Marta Dias, Patrícia Almeida

**Affiliations:** 1 Occupational Medicine, Hospitais da Universidade de Coimbra, Coimbra, PRT; 2 Occupational Medicine, Unidade Local de Saúde (ULS) do Médio Tejo, Torres Novas, PRT

**Keywords:** autonomy, burnout, health personnel, job satisfaction, leadership, occupational stress, organisational culture, work environment, workforce well-being

## Abstract

Burnout among healthcare professionals has become a critical challenge for healthcare systems worldwide. Increasing workload, administrative demands, and workforce shortages have contributed to rising levels of emotional exhaustion and professional disengagement. While burnout is often approached as an individual problem, organisational factors appear to play a central role in its development and prevention. Professional autonomy has emerged as a potentially protective factor, as greater control over clinical decision-making and work organisation is associated with improved well-being and job satisfaction. However, the benefits of autonomy depend on supportive organisational environments, adequate resources, and effective leadership. Furthermore, autonomy alone cannot offset the effects of excessive workload and chronic organisational stress. This editorial discusses the relationship between professional autonomy and burnout and argues that sustainable burnout prevention requires structural organisational changes in addition to promoting professional independence.

## Editorial

Burnout among healthcare professionals remains a major challenge for healthcare systems worldwide. Increasing workload, administrative pressure, and workforce shortages have intensified emotional exhaustion and disengagement across multiple healthcare settings. Although burnout is often discussed at the individual level, growing evidence suggests that organisational conditions play a central role in its development [1-5].

One of the most relevant organisational factors is professional autonomy. The ability to influence clinical decisions, workload organisation, and professional practice appears closely linked to professional well-being. Greater autonomy has been associated with lower burnout and improved job satisfaction among healthcare professionals [1]. At the same time, environments characterised by excessive bureaucracy and limited control over work may contribute directly to emotional exhaustion.

It is important to distinguish clinical autonomy from administrative workload control. Clinical autonomy refers to the professional's ability to make independent, evidence-based decisions regarding patient care, whereas administrative workload control relates to the capacity to influence scheduling, documentation requirements, workflow organisation, and other non-clinical demands.

Autonomy should not be interpreted simply as independence. Its impact depends largely on the context in which it is exercised. Supportive organisational environments, adequate resources, and leadership that values professional judgement appear essential for autonomy to function as a protective factor [2,3]. In practice, such environments may include adequate staffing levels, equitable workload distribution, streamlined administrative processes, access to appropriate clinical resources, and opportunities for shared decision-making. Without these conditions, the protective effects of autonomy may be substantially diminished.

Importantly, autonomy alone cannot compensate for excessive workload and chronic organisational stress. Burnout remains strongly associated with high job demands, even in professionals who report greater decision-making control [4]. In some cases, increased autonomy may even contribute to overload when responsibility expands without adequate staffing or institutional support [5].

This distinction is particularly relevant in current healthcare systems, where professionals are frequently expected to assume greater responsibility while simultaneously facing resource limitations and administrative burden. Under these conditions, promoting autonomy without improving structural working conditions risks becoming insufficient.

Healthcare leaders should recognise that autonomy is most protective when embedded within supportive organisational structures. Efforts to reduce burnout that focus primarily on individual resilience or autonomy, while leaving excessive workload and resource shortages unaddressed, are unlikely to achieve lasting results.

Ultimately, burnout should be understood not only as an individual occupational challenge but also as a reflection of organisational design. Strengthening professional autonomy represents an important component of burnout prevention strategies; however, meaningful and sustainable improvements will require broader investments in staffing, resource allocation, leadership quality, and working conditions. Future healthcare policies should therefore prioritise comprehensive organisational approaches that enable healthcare professionals to exercise autonomy within environments capable of supporting both professional performance and personal well-being.
